# Effectiveness of Anti-Gravity Treadmill Exercise After Total Knee Arthroplasty: Protocol for a Randomized Controlled Trial

**DOI:** 10.2196/59935

**Published:** 2025-02-11

**Authors:** Elina Jääskeläinen, Mikko Manninen, Heikki Hurri, Mikko Rantasalo, Yun Zhou, Hannu Kautiainen, Leena Ristolainen

**Affiliations:** 1 Laurea University of Applied Sciences Espoo Finland; 2 Orton Orthopaedic Hospital Helsinki Finland; 3 Research Institute Orton Helsinki Finland; 4 Primary Health Care Unit Kuopio University Hospital Kuopio Finland; 5 Folkhälsan Research Center Helsinki Finland

**Keywords:** total knee arthroplasty, AlterG, anti-gravity treadmill, postoperative rehabilitation, walking ability, quality of life, pain

## Abstract

**Background:**

Postoperative rehabilitation following total knee arthroplasty (TKA) varies worldwide. In Finland, patients receive guidance on safe walking and home exercises from a physiotherapist both before and after TKA. These are performed independently after surgery. However, a sedentary lifestyle is rather common among patients who have undergone TKA, with pain often limiting postoperative walking, training, and activities of daily living.

**Objective:**

This randomized controlled trial aimed to investigate the effectiveness of anti-gravity exercise, precisely the AlterG anti-gravity treadmill, on postoperative rehabilitation following TKA and to obtain new knowledge on this form of rehabilitation to better use it in the future.

**Methods:**

This randomized controlled trial study divided the patients into two groups: the intervention group and the control group. The follow-up period was 12 months. Research data were collected through questionnaires and functional tests. All patients in both groups responded to the questionnaires and participated in functional tests before surgery as well as 4 and 12 months after surgery. Patients in the intervention group exercised on the AlterG treadmill 10 times after the surgery. All the patients in this study performed the exercises as instructed when they were in the hospital. The primary outcomes were perceived pain, walking ability, and quality of life.

**Results:**

The data collection process began in 2018 and concluded in 2022, intending to obtain valuable information regarding the effect of AlterG training after TKA and determine whether it, along with traditional exercises, could be an effective form of rehabilitation that can be performed at home. We hypothesized that AlterG training would lead to faster rehabilitation, better walking quality, improved quality of life, improved physical activity, and improved overall functioning. The results of this study will be analyzed in 2025 and 2026.

**Conclusions:**

This study provides information on how AlterG training can be used in rehabilitation after TKA, further enhancing the rehabilitation program for patients undergoing TKA in general.

**Trial Registration:**

ClinicalTrials.gov NCT03904030; https://clinicaltrials.gov/study/NCT03904030

**International Registered Report Identifier (IRRID):**

DERR1-10.2196/59935

## Introduction

### Overview

Pre- and postoperative rehabilitation for total knee arthroplasty (TKA) varies worldwide [1-3]. There are guidelines for postoperative physiotherapy after TKA [4], and physiotherapy guidance for self-directed home exercises is recommended [5]. In Finland, the current care guidelines provide guidance for pre- and postoperative rehabilitation [6], but the practices associated with the rehabilitation vary among different hospitals. Previous studies have shown that both inpatient and home-based rehabilitation are effective after TKA [7] and that resistance training in water is a feasible mode of rehabilitation with a wide range of positive effects on patients undergoing TKA [8]. Reportedly, a sedentary lifestyle is rather common among patients who have undergone TKA, with pain or discomfort while standing being the greatest barrier to increasing physical activity [9]. Pain limits postoperative walking training and activities of daily living [10].

Anti-gravity exercises could help address these challenges. They enable a more objective analysis of walking, showing the entire picture of a patient’s walking problems. AlterG (AlterG, Inc., Fremont, CA), a patented compressed air technology (NASA differential air pressure technology), can be used to lighten the user’s body weight and the load of gravity with 1% accuracy, thereby enabling a less painful walking exercise compared to normal land-based training [11]. While there is limited research on the effects of AlterG training, randomized controlled trials (RCTs) with small sample sizes have been reported, particularly among patients with neurological disorders, such as cerebral palsy [12] and stroke [13,14]. These studies demonstrated that AlterG training positively affected walking speed and dynamic balance and reduced the risk of falls [12-14]. Similar results were reported in studies investigating the effects of AlterG training in rehabilitation after lower limb fractures [15,16]. Precisely, it was found that AlterG training increased muscle strength in the hip area [15] and enabled better walking [16].

A pilot study on AlterG training after TKA revealed that it increased functional ability and is thus overall a safe, useful, and effective rehabilitation method after TKA [17]. Although previous researchers [17] concluded that while functional outcomes improved over time with the use of anti-gravity gait training, further studies with a larger sample size are required to define the role of this device as an alternative or adjunct to established rehabilitation protocols [17]. Furthermore, AlterG training in the acute phase of postoperative knee rehabilitation after knee surgeries, such as TKA and anterior cruciate ligament reconstruction, demonstrated a positive effect on balance in patients experiencing increased pain in weight-bearing postures [18]. AlterG training also decreased pain, enhanced joint function, improved quality of life (QoL), and maintained thigh muscle strength gains in patients with knee osteoarthritis [19]. However, as previously mentioned, not much research has been conducted on postoperative rehabilitation including AlterG training after TKA [17,18], thereby warranting further studies in the future to obtain more knowledge regarding its effects on walking and functional capacity.

### Aims and Objectives

The exposures under investigation were the effects of anti-gravity treadmill training in postoperative rehabilitation following TKA and the added value it offered compared to traditional exercise. We hypothesized that AlterG training after hospitalization leads to faster rehabilitation, better walking quality, improved QoL, improved physical activity, and enhanced balance management compared to traditional rehabilitation methods with instructions, where patients perform the exercises independently at home. In addition, we hypothesized that the differences, in terms of the above factors, between the groups in the study—intervention group and control group—were larger in the early phase of the rehabilitation but became smaller over time. The study has been registered on ClinicalTrials.gov (NCT03904030).

This study aimed to determine the effectiveness of the AlterG anti-gravity treadmill in postoperative rehabilitation after TKA. Primary outcomes were perceived pain, walking ability, and QoL. To this end, AlterG rehabilitation and traditional postoperative rehabilitation with instructions were compared.

In detail, we aimed to measure the effects of AlterG training on:

- a patient’s walking ability and walking distance after TKA,
- a patient’s perceived QoL and functional ability after TKA,
- a patient’s perceived pain, and
- lower limb and step symmetry during gait and if it normalizes the patient’s stepping and walking.

## Methods

### Participant Selection and Sampling Strategy

Participants for this RCT study were recruited from two hospitals in the capital region of Finland: Orton Orthopaedic Hospital and Peijas Hospital, both of which are part of the HUS Helsinki University Hospital. Patients with grades 3 and 4 primary knee osteoarthritis and with a scheduled unilateral TKA were included in the study. Those with rheumatoid arthritis, who have undergone hip or knee arthroplasties within the last year, or with a BMI >40 kg/m^2^ were excluded. All eligible patients who came for a knee arthroplasty surgery at Orton Orthopaedic Hospital between 2018 and 2021 and at Peijas Hospital between 2020 and 2021 were asked to participate in the study. The patients were recruited through a nurse’s preoperative visit or a phone call made to the patients attending the surgery. The nurse then checked whether they met the inclusion and exclusion criteria; those who met the criteria were provided with written information regarding the study. The included patients were asked to provide signed consent forms (Multimedia Appendix 1), which they forwarded to the research assistant. When the patient had consented to participate in the trial, the randomization envelope was opened after the surgery. However, due to the nature of the intervention under study (rehabilitation intervention), it was not possible to blind the patients. The patients were randomly allocated either to the treatment group or the control group using a random number generator (StatTrek) by an expert who did not execute the study in practice. All the patients had free access to the available health care services during the study. Overall, 62 patients (31 in each group) were recruited for the study.

### Study Procedure

Patients in both groups underwent initial measurements 1-2 weeks before the surgery, which included questionnaires and functional tests performed by a physiotherapist. All the measurements were taken at Orton Orthopaedic Hospital. First, the patients were asked to complete the questionnaires, and functional tests were then performed. The questionnaires used were a visual analogue scale (measures perceived pain) [20], painDETECT [21], Tampa Scale of Kinesiophobia [22], RAND 36-item Health Survey 1.0 (RAND-36) [23], Oxford Knee Score [24,25], Western Ontario and McMaster Universities Osteoarthritis Index [26], Beck Depression Inventory [27], and State-Trait Anxiety Inventory [28] (Table 1). Functional tests performed were knee range of motion (ROM) [29], knee swelling [30], thigh circumference [31], single-leg stance test [32], Timed Up and Go test (TUG) [33,34], stair climbing test [35], and 6-minute walk test (6MWT) [36] (Table 1).

Patients were asked to complete the same questionnaires 6-8 weeks after surgery. The same questionnaires and functional tests were administered in the same order 4 and 12 months after the surgery at Orton Orthopaedic Hospital. This data can help obtain information regarding short-term (4 months) and long-term (12 months) changes. Then, 6 months after the surgery, the patients were sent a 6-month questionnaire regarding possible rehabilitation sessions, use of medication, and possible complications after TKA. The purpose of this questionnaire was to obtain information regarding their use of health care services and how their rehabilitation and recovery from the surgery have progressed.

**Figure 1:**
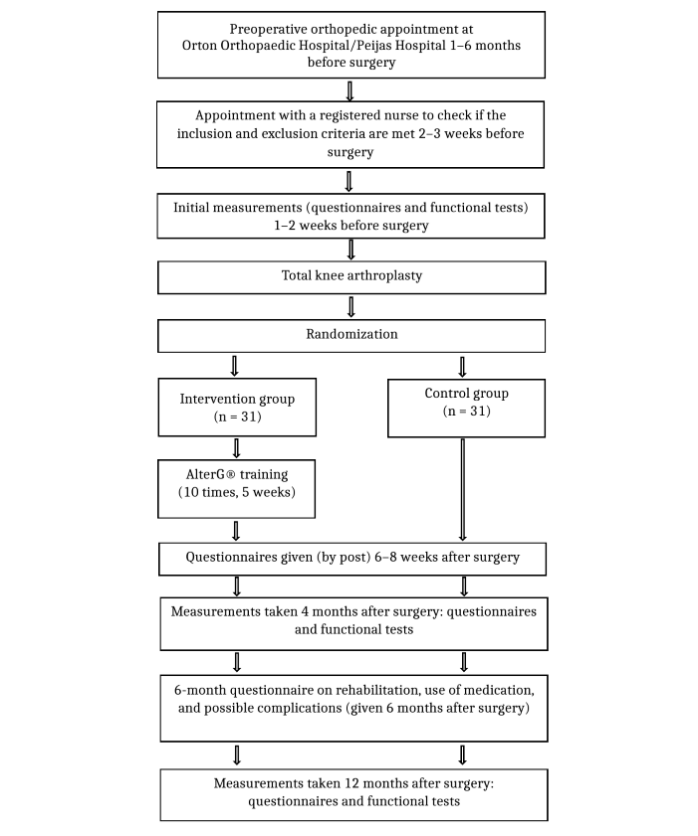
Study procedure.

After the surgery, the researcher informed each patient over the phone or face-to-face about their assigned group, either the intervention or control group. After that, the researcher booked training times on the AlterG for each member of the intervention group. Patients were sent a reminder through an SMS text message on the time reserved for them to ensure that the risk of forgetting was lower. If the time did not suit the patient, the physiotherapist was informed, and a new time was reserved accordingly. The patients in the intervention group exercised 10 times on the AlterG under the supervision of a registered physiotherapist. Both groups performed traditional postoperative exercises after TKA. The follow-up period was 12 months. Exercises began in the third week after surgery. In the third and fourth weeks after TKA, patients in the intervention group exercised twice a week on the AlterG and thrice a week in the fifth and sixth weeks considering individual variations. The AlterG training was recorded 3 out of 10 times—the first, fifth, and 10th training sessions. The recording began after the patient found a suitable walking speed and lightening. On average, the length of one recording was 2 minutes. The data obtained from the AlterG training was saved to a memory stick and then transferred to an electronic folder. The patient schedule of enrollment, interventions, and assessments are shown in Table 2. The study procedure is outlined in Figure 1.

The protocol has been developed using the SPIRIT (Standard Protocol Items: Recommendations for Interventional Trials) checklist [37] (Multimedia Appendix 2).

**Table 1:** Questionnaires and functional tests.

Measurement				Explanation
**Questionnaires**				
	**Pain**			
		Severity of pain: VAS^a^ [20]	The scale ranges from 0 to 100, where 0 indicates no pain at all and 100 indicates the worst possible pain.	
		Neuropathic pain: painDETECT [21]	The screening questionnaire identifies neuropathic components in patients with pain.	
		Fear of movement: TSK^b^ [22]	The screening questionnaire detects fear of movement due to pain.	
		OKS^c^ [24,25]	The 12-item questionnaire assesses knee pain and functionality.	
		WOMAC^d^ [26]	The disease-specific questionnaire explores the effects of osteoarthritis treatment intervention.	
	**Quality of life**			
		RAND-36^e^ [23]	The questionnaire explores well-being and functional ability across eight dimensions: general health perceptions, physical functioning, emotional well-being, social functioning, energy, bodily pain, role functioning/physical, and role functioning/emotional.	
	**Depression and anxiety**			
		BDI-21^f^ [27]	The 21-item questionnaire measures depression.	
		STAI^g^ [28]	The 40-item questionnaire measures trait and state anxiety.	
**Functional tests**				
	Knee ROM^h^ in degrees (°) [29]		Knee ROM (flexion and extension) is measured using a goniometer.	
	Knee swelling (cm) [30]		Knee joint swelling is measured using a tape measure at the popliteal.	
	Thigh circumference (cm) [31]		The thigh circumference is measured using a tape measure 15 cm above the upper edge of the patella.	
	Single-leg stance (s) [32]		The balance test assesses static balance and upright posture control on a support surface narrower than a normal stance.	
	TUG^i^ (s) [33,34]		The functional test measures an individual’s mobility/ability to move.	
	Stair climbing test (s) [35]		The functional test measures the functionality of the lower extremities and balance control. Here, the test was systematically conducted on the same staircase, which had 11 steps. The depth of one step was 34 cm, and the height was 14.5 cm.	
	6MWT^j^ (m) [36]		The functional test measures an individual’s endurance and walking ability.	

^a^VAS: visual analogue scale.

^b^TSK: Tampa Scale of Kinesiophobia.

^c^OKS: Oxford Knee Score.

^d^WOMAC: Western Ontario and McMaster Universities Osteoarthritis Index.

^e^RAND-36: RAND 36-item Health Survey 1.0.

^f^BDI-21: Beck Depression Inventory.

^g^STAI: State-Trait Anxiety Inventory.

^h^ROM: range of motion.

^i^TUG: Timed Up and Go test.

^j^6MWT: 6-minute walk test.

**Table 2:** Patient schedule of enrollment, interventions, and assessments.

			Enrollment		Study period															
					Initial assessment		Allocation		Post-allocation											Closeout
			–2 to –3 weeks		–1 to –2 weeks		0 weeks		3-6 weeks		6-8 weeks		4 months		6 months		12 months		12 months	
**Enrollment**																				
	Eligibility screen	✓																		
	Informed consent	✓																		
	Allocation					✓														
**Interventions**																				
	Intervention (AlterG)							✓^a,b^		✓^b^										
	Control group (traditional exercises)							✓^b^		✓^b^										
**Assessments**																				
	Questionnaires (VAS^c^, PainDETECT, TSK^d^, RAND-36^e^, OKS^f^, WOMAC^g^, BDI-21^h^, and STAI^i^)			✓						✓		✓				✓		✓		
	Functional tests (ROM^j^, knee swelling, thigh circumference, single-leg stance, stair climbing test, and 6MWT^k^)			✓								✓				✓		✓		
	6-month questionnaire													✓						

^a^AlterG training (10 times).

^b^Traditional home exercises that the patient was instructed to perform in the hospital after the total knee arthroplasty.

^c^VAS: visual analogue scale.

^d^TSK: Tampa Scale of Kinesiophobia.

^e^RAND-36: RAND 36-item Health Survey 1.0.

^f^OKS: Oxford Knee Score.

^g^WOMAC: Western Ontario and McMaster Universities Osteoarthritis Index.

^h^BDI-21: Beck Depression Inventory.

^i^STAI: State-Trait Anxiety Inventory.

^j^ROM: range of motion.

^k^6MWT: 6-minute walk test.

### Postoperative Exercises

Furthermore, all the patients (intervention and control groups) in the study independently performed the postoperative exercises instructed by the hospital’s physiotherapists. The exercises were aimed to improve knee ROM and quadriceps and hamstring muscle strength and to stimulate venous circulation. Patients also received guidance on walking with crutches. They were instructed to perform home exercises for approximately 2 months after surgery. However, the researchers do not have information on how actively the patients were engaged with home exercises. The home exercises are described in Table 3.

**Table 3:** Traditional home exercises.

Exercise	Description	Aim
Knee flexion (sitting/supine position)	Sitting on a chair or in the supine position, the patient flexes the knee as much as possible, sliding the sole of the foot backward on the ground. The patient keeps the knee flexed for a while and then returns the lower limb to the starting position, sliding the sole of the foot forward.	To increase knee mobility in the direction of flexion
Knee flexion (standing position)	In the standing position, the patient brings the operated foot on a stair and shifts the weight forward, stretching the knee in the direction of flexion.	To increase knee mobility in the direction of flexion
Quadriceps strengthening (sitting/supine position)	In the sitting or supine position on a bed, the patient extends the knee and presses it against the platform or a towel roll by activating the quadriceps muscles. When performing this in the supine position, at the end of the movement, the patient can lift the straight leg off the ground. Quadriceps strengthening can also be done by sitting on a chair and lifting the straight leg off the ground.	To activate and strengthen the quadriceps muscles and the end-extension of the knee
Ankle pump movements (flexion and extension)	In the supine position, the patient flexes and extends the ankles in turns.	To stimulate and enhance venous blood circulation
Dynamic calf stretching	In the standing position, the patient performs a dynamic calf stretch, taking a step backward with the operated leg and pressing the heel on the ground while extending the knee and then lifting the heel off the ground and letting the knee flex.	To stretch the calf muscles
Hamstring stretching	In the sitting position and with the operated leg straight on the bed, the patient bends the upper body forward and stretches the hamstring muscles.	To stretch the hamstring muscles
Toe raises	In the standing position, the patient takes support from the back of the chair with their hands and rises to their toes and then returns to the starting position.	To activate the calf muscles
Walking with crutches	The patient is instructed on how to walk with crutches after the surgery, using either three-point walking or alternating walking, or with only one crutch. The patient is also instructed to walk on stairs with a crutch/crutches.	To guide the patient on how to use crutches after the surgery

### Outcome Assessment and Measurements

The primary outcomes were walking ability measured with the 6MWT, health-related QoL measured with RAND-36, and perceived pain measured with VAS. All outcomes were measured before the TKA and 4 and 12 months after the TKA. In addition to this, the patients were asked to complete the same questionnaires 6-8 weeks after the TKA. The initial measurements were performed 1-2 weeks before the TKA. Depending on the patient, it took a total of 1.5-2 hours to complete the questionnaires and execute the functional tests. The same measurements were performed 4 and 12 months after the surgery, and the time required for these was the same as in the initial measurements (1.5-2 hours). Regarding the functional tests, the test scores were not calculated within the testing situation; however, the physiotherapist noted the measurement results on the test form, which were then saved in an electronic format. The results obtained from the tests were compared throughout the study between the patient’s own results and between the intervention and control groups; no comparisons were made with the general reference values of the tests. The knee ROM and swelling, thigh muscle circumference, TUG, stair climbing test, and 6MWT were performed only once. The tests were performed twice only in the static balance test, and the best test result was recorded (the maximum time was 60 seconds).

All the measures of the study are presented in Table 1. During the AlterG training, the weight-bearing symmetry, step length, stance time symmetry, and cadence during walking were measured (Textbox 1).

**AlterG measurements.**

**Weight-bearing symmetry**

- Weight-bearing symmetry gives information about the symmetry of the stance phase. In pathological situations, the stance phase is shorter on the weaker side [38].

**Step length symmetry**

- Step length is the distance from the back of one heel to the back of the other heel [38].

**Stance phase symmetry**

- Stance phase is the period when the foot is on the ground [38].

**Cadence (steps/min)**

- Cadence is the number of steps taken per minute [38].

### Data Collection and Statistical Analysis

We estimated that a difference of 30 m in the 6MWT between the two groups would represent a clinically relevant difference [39,40]. To identify such a difference with 2-sided testing (α=.05 and power of 85%), the study required 31 participants in each group, with the assumption of 20% loss to follow-up.

The analysis will use the intention-to-treat principle, which will include all randomized patients. Summary statistics will be described using mean and SD, median and IQR, or numbers and percentages. Statistical comparison between the groups will be performed using the *t* test, Mann-Whitney test, *χ*^2^ test, or Fisher-Freeman-Halton test, as appropriate. A linear mixed model or generalized estimating equation model with appropriate distribution and link function for repeated measurements will be used for analysis. In case of a violation of the test assumptions, a bootstrap-type method or Monte Carlo method will be used. Normal distributions will be graphically evaluated using the Shapiro-Wilk W test. Stata 18 (StataCorp LP) will be used for the analysis. Access to data will be granted to the research team of this study.

### Ethical Considerations

The study protocol was approved by the ethics committee of the Hospital District of Helsinki and Uusimaa in 2017 (HUS/3117/2017). Helsinki University Hospital, Peijas Hospital, was also included in the study in 2020, and updated ethical permission and research permission were received from HUS (HUS 234/2020). Good research ethics practices were maintained in the study in accordance with the Declaration of Helsinki. Before participating in the study, every patient was given an information letter that would help them decide whether they wanted to participate in the study; once the decision was made, they were asked to sign a written consent form. The patients had the right to discontinue the trial whenever they wanted without giving any reason. However, the data collected before the discontinuation can be used for research purposes (Multimedia Appendix 1).

There was no inclusion of vulnerable groups such as children, prisoners, or individuals with mental disability. No participant reimbursement was provided to prevent economic factors from impacting the recruitment process.

If there were any changes to the protocol, the ethics committee and other relevant parties were informed.

### Ensuring Data Quality

Anonymity and confidentiality were ensured by using numerical codes for the participants. Only the research group members had access to the participants’ names. Data protection and storage security were ensured by storing the participant information and questionnaires in a locked cabinet at Orton Orthopaedic Hospital. Data were securely stored with electronic passwords on the hospital’s server.

## Results

The data collection began in 2018 and concluded in 2022. This study aimed to obtain valuable information on the effect of AlterG training after TKA. AlterG, along with traditional exercises, could be an effective form of rehabilitation that can be performed at home. We hypothesized that AlterG training leads to faster rehabilitation, better walking quality, improved QoL, improved physical activity, and improved overall functioning. At baseline, there were 62 participants in the study, with 31 in each group. Of these, 35 (56%) were women, and 27 (44%) were men, with a mean age of 66 (SD 7) years. The results of this study will be analyzed in 2025 and 2026. Results from this study will be submitted for publication in peer-reviewed international scientific journals and presented at scientific meetings.

## Discussion

### Expected Findings

The expected findings were related to walking ability, health-related QoL, and perceived pain. The expectations were that pain would decrease, walking distance would be longer, and health-related QoL would improve. In addition, we expected that the differences between the groups would be larger in the short term and eventually level out in 12 months.

### Comparisons With Prior Work

Studies on postoperative rehabilitation after TKA including AlterG training are scarce [17,18]. To our knowledge, Bugbee et al [17] were the only ones who investigated the effects of AlterG training in patients who underwent TKA in an RCT. Hence, the present study was conducted to further investigate and obtain more information on the effects of AlterG training in patients who underwent TKA.

### Limitations and Strengths of the Protocol

One of the limitations of this study was the risk of dropouts when patients heard that they were not included in the intervention group. Second, not all patients were committed to the 12-month follow-up. Third, the implementation of the study coincided with the COVID-19 pandemic, impacting the progress of the study and leading to dropouts.

However, this study also has some strengths. First, this is an RCT study. Second, to the best of our knowledge, there has been only one pilot and feasibility study [17] that directly focused on this issue thus far. With this relatively novel study, it is possible to collect more valuable information on how anti-gravity exercise can be used in rehabilitation after TKA. Third, it investigated aspects of a patient’s physical functioning, perceived QoL, and pain rather extensively, thereby providing an opportunity to control various sources of bias such as state of depression and neuropathic pain. Lastly, the study design was planned in a multi-professional manner.

### Study Significance and Feasibility

The results of this study provided information on how AlterG can be used in rehabilitation after TKA. With this knowledge, hospitals may potentially develop and enhance the rehabilitation program for patients who undergo knee arthroplasty. It is also possible to use the research results more widely with other patient groups, such as those with lower limb problems and athletes.

### Conclusion

The results of this study provided information on how AlterG training can be used in rehabilitation after TKA. This information may enable the enhancement and development of a rehabilitation program for patients undergoing TKA.

## Appendix

Multimedia Appendix 1 Consent form.

Multimedia Appendix 2 CONSERVE (CONSORT and SPIRIT Extension for RCTs Revised in Extenuating Circumstances)–SPIRIT (Standard Protocol Items: Recommendations for Interventional Trials) checklist.

## Abbreviations

QoL: quality of life
RAND-36: RAND 36-item Health Survey 1.0
RCT: randomized controlled trial
ROM: range of motion
SPIRIT: Standard Protocol Items: Recommendations for Interventional Trials
TKA: total knee arthroplasty
TUG: Timed Up and Go test
6MWT: 6-minute walk test

## Data Availability

The datasets generated or analyzed during this study are available from the corresponding author upon reasonable request.
