# Supplementary material for: Effectiveness of Anti-Gravity Treadmill Exercise After Total Knee Arthroplasty: Protocol for a Randomized Controlled Trial
Source: JMIR Res Protoc. 2025 Feb 11;14:e59935. doi: 10.2196/59935 (PMC11862769; doi:10.2196/59935)
Supplement: Multimedia Appendix 1 [file resprot_v14i1e59935_app1.pdf]

**CONSENT**

I have received, read and understood the information leaflet concerning the **Unweighted rehabilitation** study. This leaflet has provided me with an adequate clarification regarding the study and the associated data collection, processing and disclosure.

**I consent** to a study investigating the effect of rehabilitation on physical and mental functional ability following total knee arthroplasty. I consent to the collection of information about me that is essential to the study in Tieteellinen tutkimus Orton's study data file, and I give consent for the information about me obtained from total knee arthroplasty, tests carried out by a physiotherapist, and questionnaires to be made available to researchers to the extent necessary for the study.

All information collected about me during the study will be processed confidentially. The information collected in the study will be encrypted in such a way that it will not be possible to discern a participant's identity without a decryption code. The decryption code is stored closed in the researching doctor's archives or in the archives of Tieteellinen tutkimus Orton.

I understand that my participation in this study is completely voluntary. I have the right at any time to discontinue my participation in the study or withdraw my consent without explanation. Withdrawing my consent will result in any negative consequences for me and will not affect my status as a healthcare customer. I am aware that information collected prior to my withdrawal from the study or the withdrawal of my consent will be used as part of the study data to the extent necessary in order to avoid any distortion of the study results.

A copy of this consent form will be given to the participant; the original copy will remain in the researcher's archives.

Date: ................................................ / 20..

Signature of participant:.......................................................

Name in print:........................................................................

Personal ID number:.........................................................................

Address:...............................................................................................

Phone number: .......................................................................
(Please provide your phone number if you are happy for us to contact you to clarify anything that may be unclear)

Consent received by: .......................................................................

Name in print: ........................................................................................

Date received: .....................................................................
